# An enhanced GhostNet model for emotion recognition: leveraging efficient feature extraction and attention mechanisms

**DOI:** 10.3389/fpsyg.2024.1459446

**Published:** 2025-04-09

**Authors:** Jie Sun, Tianwen Xu, Yao Yao

**Affiliations:** ^1^Psychological Development Guidance Center, School of Educational Sciences, Quanzhou Normal College, Quanzhou, China; ^2^Professional College of Arts and Tourism, Hyogo Public University Corporation, Hyogo, Japan

**Keywords:** emotion recognition, decision-making, GhostNet, Transformer Encoder, dual attention mechanism, human-computer interaction

## Abstract

Emotion recognition plays a crucial role in understanding decision-making processes, as emotional stimuli significantly influence individuals' choices. However, existing emotion recognition systems face challenges in handling complex natural environments, diverse emotional expressions, and limited data availability, hampering their effectiveness and widespread adoption. To address these issues, we propose an Enhanced GhostNet with Transformer Encoder (EGT) model that leverages deep learning techniques for robust emotion recognition through facial expressions. The EGT model integrates GhostNet's efficient feature extraction, the Transformer's ability to capture global context, and a dual attention mechanism to selectively enhance critical features. Experimental results show that the EGT model achieves an accuracy of 89.3% on the RAF-DB dataset and 85.7% on the AffectNet dataset, outperforming current state-of-the-art lightweight models. These results indicate the model's capability to recognize various emotional states with high confidence, even in challenging and noisy environments. Our model's improved accuracy and robustness in emotion recognition can enhance intelligent human-computer interaction systems, personalized recommendation systems, and mental health monitoring tools. This research underscores the potential of advanced deep learning techniques to significantly improve emotion recognition systems, providing better user experiences and more informed decision-making processes.

## 1 Introduction

The role of emotional stimuli in the decision-making process is a significant research area in psychology and cognitive science (Juárez-Varón et al., 2023). Emotions influence not only individuals' daily life decisions but also play a crucial role in fields such as business, medicine, and human-computer interaction. Understanding how emotions influence decision-making behavior through facial expressions is of great importance for enhancing the intelligence and effectiveness of various intelligent systems (Zhao et al., 2022). However, current emotion recognition and decision prediction systems still face numerous challenges when dealing with complex natural environments. The diversity of emotional expressions, environmental interference, data scarcity, and the subjectivity and complexity of emotional states are pressing issues that need to be addressed in current research. These challenges limit the widespread adoption and effectiveness of emotion recognition systems in practical applications (Morelli et al., 2022). In recent years, deep learning technology has made significant progress in image processing and pattern recognition. Its powerful feature extraction and representation capabilities have made deep learning one of the core methods for facial expression recognition research. Convolutional Neural Networks (CNNs) have shown outstanding performance in image classification and feature extraction. Through multi-layer convolution and pooling operations, they can effectively extract distinguishable emotional features (Zhou et al., 2022). Recurrent Neural Networks (RNNs) and their variants, Long Short-Term Memory Networks (LSTMs), have demonstrated strong capabilities in handling time-series data, making them suitable for capturing the dynamic characteristics of emotions over time (Elliott et al., 2023). Additionally, the recently emerged Transformer models, which capture global information through self-attention mechanisms, have excelled in many tasks (Singh et al., 2022). These technical advantages of deep learning models provide unique benefits in handling complex emotional data and improving the accuracy and robustness of emotion recognition.

The application of deep learning technology in the field of facial expression recognition has not only improved the accuracy of emotion recognition but also promoted in-depth research on the relationship between emotions and decision-making behavior. For instance, emotion classification models can assist in predicting users' decision tendencies under different emotional states, optimizing the responsiveness of personalized recommendation systems and intelligent interaction systems (Ge et al., 2022). These advancements are particularly beneficial in practical applications such as human-computer interaction (HCI) and mental health monitoring. In HCI, accurate emotion recognition can facilitate more natural and responsive interactions, improving user experience in systems like virtual assistants and interactive robots. In mental health monitoring, emotion recognition systems can be used to detect early signs of emotional distress or mental health issues, providing timely interventions and personalized support. Understanding the impact of emotions on the decision-making process can also be applied to mental health monitoring and intervention systems, helping to detect and intervene in potential psychological issues promptly (Bisogni et al., 2022). Deep learning technology, through its end-to-end learning approach, reduces dependence on manual feature engineering, enhancing the automation level of models. It can handle large amounts of unstructured data such as images, videos, and audio, making it suitable for complex and variable natural environments (Umer et al., 2022). By extracting multi-level features and adaptive optimization of deep learning networks, deep learning surpasses traditional methods in the accuracy and robustness of emotion recognition. In conclusion, deep learning technology provides powerful tools for facial expression recognition in the study of emotional stimuli and decision-making mechanisms. It overcomes many limitations of traditional methods in feature extraction and data processing, driving rapid development in related research fields. In the future, facial expression recognition systems are expected to have broader applications in fields such as HCI and healthcare, contributing to improved interaction and mental health monitoring. In the future, with the continuous advancement of deep learning technology and the diversification of data acquisition, facial expression recognition systems will play a more significant role in more application scenarios, further revealing the profound impact of emotions on the decision-making process.

In recent years, significant progress has been made in the study of emotion recognition and decision-making behavior, with many studies dedicated to exploring the impact of facial expression recognition on decision-making processes. For instance, one study utilized Convolutional Neural Networks (CNN) to recognize facial expressions and examined the influence of emotions on consumer purchasing decisions. This study, set against the backdrop of e-commerce platforms aiming to optimize recommendation systems by analyzing users' emotional states to boost sales, employed a Deep CNN model, specifically the VGG-16 model, trained and tested on a large facial expression dataset (Gupta et al., 2023). The goal was to provide personalized product recommendations through real-time emotion recognition. The results indicated an emotion recognition accuracy of 85%, significantly enhancing the click-through and conversion rates of the recommendation system. However, the study noted a decline in recognition accuracy in noisy environments, affecting the stability of practical applications.

Another related study focused on utilizing Long Short-Term Memory Networks (LSTM) to capture the dynamic impact of emotional changes on decision-making processes (Febrian et al., 2023). This study, set against the backdrop of investment decisions in financial markets, aimed to predict market trends by analyzing investors' emotional fluctuations. The study employed an LSTM model combined with an emotion classifier to model and predict investors' emotional changes under different market conditions. The objective was to provide more accurate market forecasts and investment advice by capturing the time-series characteristics of emotional fluctuations. Experimental results showed a 15% improvement in prediction accuracy in volatile market environments compared to traditional methods. However, the study highlighted the challenges of using LSTM models in terms of computational complexity, which limited their applicability in real-time scenarios. Additionally, another study introduced Generative Adversarial Networks (GAN) to enhance the diversity of emotion recognition data, aiming to improve the generalization ability of models (Guo et al., 2023). Set in the field of affective computing, this study aimed to generate more high-quality emotional expression data to compensate for the lack of real data. The study employed a GAN-based model to generate realistic facial expression images for training emotion recognition models. The goal was to improve the performance of emotion recognition models in various scenarios through data augmentation techniques. Experimental results indicated that the data generated by GAN significantly improved the accuracy of emotion recognition models on small sample datasets by 20%. However, the study noted that GAN-generated images still exhibited unnatural phenomena in some complex expressions, potentially affecting the accuracy of recognition models. Moreover, another study combined the Transformer architecture to capture global emotional features, aiming to improve the accuracy and stability of emotion recognition (Liang et al., 2023). This study, set against the backdrop of intelligent monitoring systems, aimed to provide early warnings of potential dangerous behaviors through precise emotion recognition. The study employed the Transformer model, which uses self-attention mechanisms to analyze and capture global information from facial expressions in videos. The objective was to monitor and warn of abnormal behaviors in real-time by analyzing emotions across consecutive video frames. Experimental results showed that the Transformer model outperformed traditional CNN and RNN models in both accuracy and stability of emotion recognition, especially in handling long video sequences. However, the study pointed out that the high computational resource requirements of the Transformer model might limit its application in resource-constrained environments.

Despite the progress made in emotion recognition and decision analysis, several challenges remain. One major issue is the robustness of emotion recognition in noisy and complex environments. Additionally, the high computational resource requirements of deep learning models continue to limit their widespread application in real-time and mobile environments. Addressing these challenges is crucial for enhancing the effectiveness and adoption of emotion recognition systems.

Based on the shortcomings of the aforementioned studies, we propose an enhanced GhostNet with Transformer Encoder (EGT) network. The motivation for this new model arises from the current methods' lack of robustness in handling emotion recognition in complex environments. Our aim is to overcome this challenge by integrating efficient feature extraction, a Transformer encoder, and dual attention mechanisms. The GhostNet feature extraction module, with its unique structural design, achieves efficient feature extraction while reducing computational load and memory usage, significantly enhancing the model's computational efficiency. The Transformer encoder is employed to capture global information, while the dual attention mechanism selectively enhances important features, providing higher accuracy and robustness in dealing with complex emotional expressions. The EGT model, through the integration of efficient feature extraction, the Transformer encoder, and dual attention mechanisms, introduces significant technical innovations. It demonstrates outstanding effectiveness in addressing key issues in current emotion recognition tasks. The EGT model not only improves the accuracy and robustness of emotion recognition but also significantly enhances the model's efficiency in real-time applications. This provides new methods and tools for improving intelligent systems that require accurate emotion recognition in challenging environments.

Based on our in-depth research on emotion recognition and decision prediction, our main contributions are as follows:

- We propose an enhanced GhostNet with Transformer Encoder (EGT) model, which effectively improves the accuracy and robustness of emotion recognition by combining GhostNet's efficient feature extraction, the Transformer encoder's ability to capture global information, and a dual attention mechanism for selective feature enhancement.
- We design an innovative dual attention mechanism, including channel attention and spatial attention, which selectively enhances important features in both the feature channel and spatial dimensions. This improves the model's ability to recognize emotions in complex environments.
- We validate the superior performance of the EGT model through extensive experiments on multiple emotion recognition datasets. These experiments demonstrate the model's effectiveness in handling complex emotional expressions, providing a robust solution for studying emotion recognition in challenging environments.

The rest of this paper is structured as follows: Section 2 reviews related work in emotion recognition. Section 3 outlines the methodology of the proposed EGT model. Section 4 describes the experimental setup and data used. Section 5 discusses the results and analysis. Finally, Section 6 concludes with a summary of findings, limitations, and future research directions.

## 2 Related work

### 2.1 Application of multimodal data fusion technology in emotion recognition

In recent years, multimodal data fusion technology has gained widespread attention in the field of emotion recognition. Emotional expression is typically multimodal, involving facial expressions, voice, body movements, and physiological signals (Ezzameli and Mahersia, 2023). By combining data from different modalities, it is possible to recognize an individual's emotional state more comprehensively and accurately. This multimodal fusion approach overcomes the limitations of single-modal emotion recognition, significantly improving performance and robustness (Wang et al., 2024).

Multimodal data fusion techniques mainly include feature-level fusion and decision-level fusion. Feature-level fusion involves combining data features from different modalities during the feature extraction phase to form a comprehensive feature vector (Chango et al., 2022). This method requires standardizing features from each modality to eliminate differences in scale, followed by feature concatenation or dimensionality reduction techniques such as Principal Component Analysis (PCA) to create a unified feature vector for input into a classifier for emotion recognition. Feature-level fusion can capture the potential relationships between features from different modalities, which enhances the accuracy of emotion recognition. For example, in the fusion of facial expression and speech emotion recognition, CNNs can extract facial expression features, while LSTMs are used to extract speech features, and these features are then fused to improve overall emotion recognition performance (Liu et al., 2023). Decision-level fusion, on the other hand, involves independently recognizing emotions from each modality and then combining the recognition results. Common fusion strategies include weighted voting, Bayesian methods, and Dempster-Shafer theory (Atmaja et al., 2022; Wu and Li, 2023). Weighted voting assigns different weights to the recognition results of each modality and makes a decision based on these weights. Bayesian methods compute posterior probabilities for each modality and select the most probable emotional state. Dempster-Shafer theory combines evidence from different modalities to obtain more reliable decision results (Sharma et al., 2023). The advantage of decision-level fusion lies in its flexibility and robustness, as it effectively combines recognition results from different modalities to improve overall emotion recognition performance.

The use of advanced neural network techniques in multimodal emotion recognition has achieved considerable advancements. CNNs and RNNs have shown excellent performance in feature extraction, capable of automatically extracting and fusing multimodal features. For example, models that combine CNNs and LSTMs can handle both image and temporal data simultaneously, achieving more efficient emotion recognition (Gandhi et al., 2023). Generative models such as Generative Adversarial Networks (GANs) and Variational Autoencoders (VAEs) enhance model generalization and robustness through data augmentation and feature extraction (Kumar et al., 2022). The introduction of attention mechanisms helps models more effectively select and weight features from different modalities during feature-level fusion. The application of multimodal data fusion technology extends beyond emotion recognition to other areas of affective computing, such as emotion prediction and emotional interaction. In emotion prediction, multimodal fusion technology can combine historical emotional data with current emotional states to more accurately predict an individual's emotional changes (Kumar et al., 2024; Ning et al., 2024). In emotional interaction, multimodal fusion technology can achieve more natural and intelligent human-computer interaction by comprehensively analyzing users' facial expressions, voice, and body movements.

Despite the significant potential of multimodal data fusion technology in emotion recognition, several challenges remain in its application. First, the collection and annotation of multimodal data are costly, and the quality and temporal synchronization of data from different modalities may vary (Wang S. et al., 2023). Second, the high dimensionality and complexity of multimodal data increase the difficulty of model training, requiring more efficient feature extraction and fusion algorithms. Additionally, the high variability in emotional expression among individuals poses a challenge in handling these differences in multimodal emotion recognition. In summary, multimodal data fusion technology significantly enhances the accuracy and robustness of emotion recognition by combining information from different modalities, providing strong support for building more intelligent and humanized affective computing systems. As application demands and technology continue to evolve, ongoing exploration and optimization of multimodal data fusion methods and technologies are necessary to address the challenges in practical applications.

### 2.2 Applications of self-supervised learning and transfer learning in emotion recognition

Self-supervised learning and transfer learning are two critical technologies in the field of emotion recognition, showing significant potential in enhancing model performance and reducing data dependency in recent years (Yu et al., 2024).

Self-supervised learning is a method that trains models using unlabeled data. Unlike traditional supervised learning, which relies on a large amount of labeled data, self-supervised learning designs pre-training tasks to learn feature representations from unlabeled data, which are then used for downstream tasks (Zhao et al., 2023). In emotion recognition, self-supervised learning can extract useful features from a large volume of unlabeled facial expressions, speech, or other emotional data through pre-training tasks such as rotation prediction, occlusion reconstruction, or video frame sorting (Wang X. et al., 2023). These pre-training tasks help models capture the intrinsic structure and patterns of the data, improving the quality of feature representation. When these self-supervised pre-trained features are used for emotion recognition, they can significantly enhance model performance, especially when labeled data is scarce. Transfer learning, on the other hand, involves applying knowledge learned from a pre-trained model on one task to another related task. By pre-training models on large-scale datasets and then fine-tuning them for specific emotion recognition tasks, transfer learning can effectively leverage the knowledge of pre-trained models to improve the accuracy and robustness of emotion recognition (Chaudhari et al., 2023; Huang et al., 2023). For example, Convolutional Neural Networks (CNNs), initially trained on extensive image datasets like ImageNet, can be further adjusted to meet the particular requirements of facial expression recognition tasks (Latif et al., 2022). Similarly, Long Short-Term Memory Networks (LSTM) or Transformers pre-trained on large-scale speech datasets can be fine-tuned for speech emotion recognition tasks, thus improving model performance.

The combination of self-supervised learning and transfer learning shows significant advantages in emotion recognition. By pre-training feature representations through self-supervised learning and then transferring these representations to emotion recognition tasks for fine-tuning, model performance can be improved while reducing the need for labeled data (Morais et al., 2022). For example, in facial expression recognition, self-supervised learning can be used to learn rotation prediction tasks from a large amount of unlabeled facial images, and then the learned features can be transferred to labeled emotion recognition tasks for fine-tuning, thereby significantly improving recognition accuracy. Transfer learning can also help address the issue of data distribution inconsistency in emotion recognition. By pre-training on large-scale datasets related to the target task, the model can learn more extensive feature representations that better adapt to the data distribution of the target emotion recognition task (Li and Xiao, 2023). Self-supervised learning and transfer learning also show potential in addressing the issue of individual variability in emotion recognition. The variability in emotional expression among individuals is a major challenge in emotion recognition (Chen et al., 2023). Self-supervised learning can derive generalized feature representations from extensive collections of unlabeled data obtained from various individuals, capturing commonalities between them (Wu et al., 2023). Fine-tuning with transfer learning on specific individuals' data can then make the model better adapt to individual differences, thereby improving the accuracy of emotion recognition.

Despite the significant potential of self-supervised learning and transfer learning in emotion recognition, challenges remain. For instance, the design of pre-training tasks in self-supervised learning needs careful consideration to ensure that the learned features are useful for downstream emotion recognition tasks (Rafiei et al., 2022). Additionally, the fine-tuning process in transfer learning needs to balance the knowledge of the pre-trained model with the specific needs of the target task to avoid overfitting or underfitting.

## 3 Method

### 3.1 Overview of our network

This study aims to improve the accuracy and robustness of emotion recognition and decision prediction by proposing an enhanced GhostNet model, named Enhanced GhostNet with Transformer Encoder (EGT). The EGT model consists of three main components: the GhostNet feature extractor, the Transformer encoder, and a dual attention mechanism. The GhostNet feature extractor is used to extract fundamental features from raw data using lightweight convolution operations, which significantly reduce computational cost and parameter count while maintaining high accuracy. Its efficiency and simplicity enable the model to process large-scale data more quickly, providing high-quality fundamental features for subsequent modules. The Transformer encoder enhances the expressiveness of these features by capturing global context. By simultaneously attending to all parts of the input sequence, the Transformer encoder offers a comprehensive understanding of the data's structure and context, resulting in more stable and accurate handling of complex emotional data. The dual attention mechanism, comprising channel attention and spatial attention, selectively enhances important feature representations. Channel attention focuses on different channels of the feature map, while spatial attention addresses different spatial locations within the feature map. By combining these two attention mechanisms, the model effectively highlights critical features and suppresses irrelevant or redundant information, thereby improving overall recognition and prediction performance.

**Figure 1:**
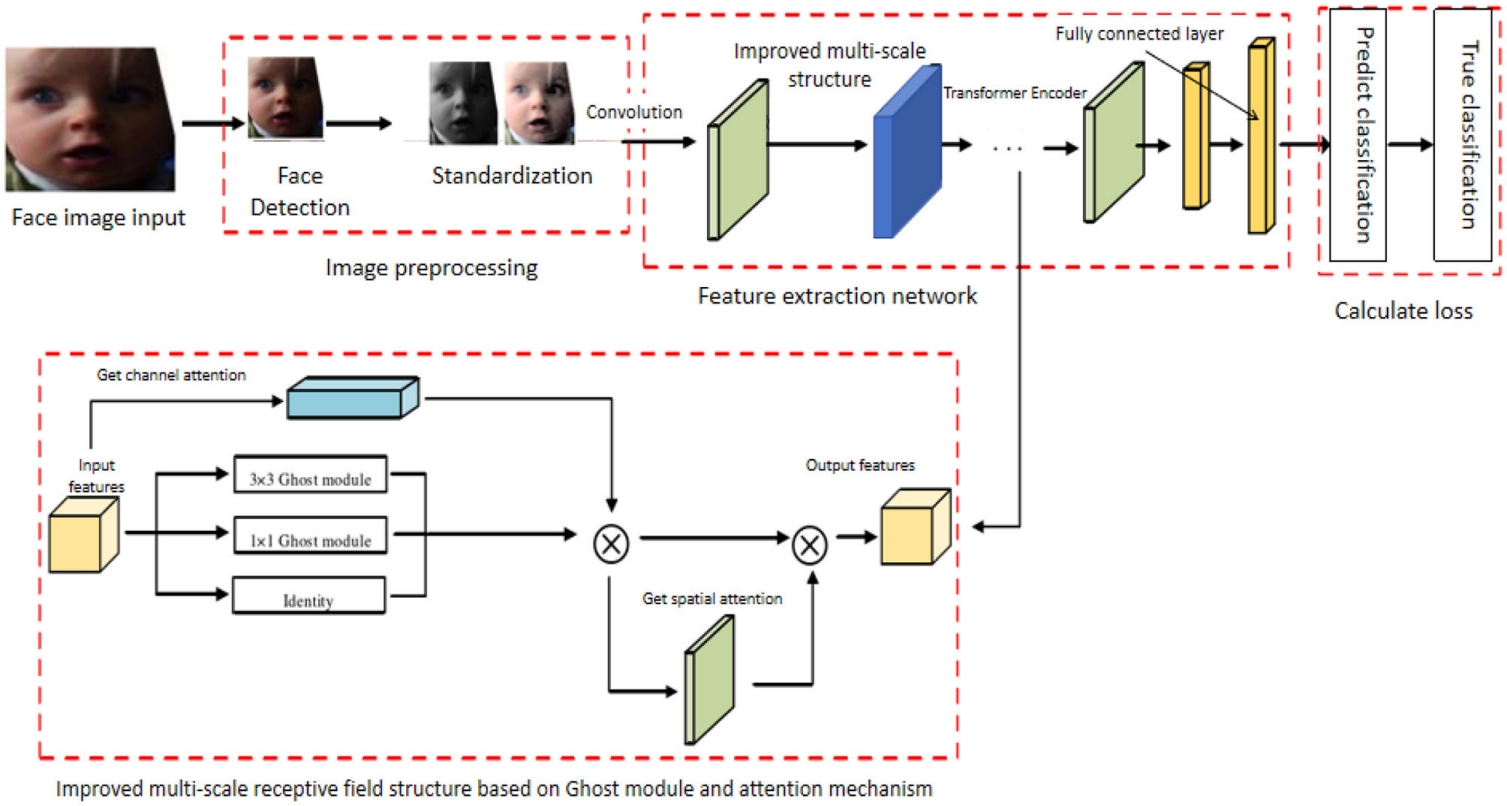
Overall architecture of the EGT network, illustrating the stages of image preprocessing, feature extraction using GhostNet, Transformer Encoder, attention mechanisms, and final emotion classification. Facial images are adapted with permission from the RAF-DB dataset (Li et al., https://www.kaggle.com/datasets/shuvoalok/raf-db-dataset).

During the construction of the EGT network, data preprocessing is performed first, extracting images and sequence data from the dataset, normalizing image data, and applying various methods to enhance the training dataset. Next, the GhostNet feature extractor is built to extract fundamental features from the preprocessed image data using lightweight convolution operations, and the extracted feature maps are input into the Transformer encoder. In the Transformer encoder section, multiple self-attention heads are utilized to capture overall contextual information. Subsequently, channel and spatial attention mechanisms are added to enhance the representation of critical features. Finally, the enhanced features are input into a fully connected layer or other classifiers for the final classification of emotional states, outputting the emotion recognition results. Figure 1, where each component's role and interaction are clearly illustrated, provides a comprehensive view of the model's structure and workflow.

The EGT model has significant advantages and innovations in emotion recognition research. First, the GhostNet feature extractor, through lightweight convolution operations, significantly reduces computational cost and parameter count, improving the model's processing speed and efficiency, allowing it to quickly process large-scale data. Second, the Transformer encoder, with its multi-head self-attention mechanism, effectively captures global information in the input sequence, enhancing feature expressiveness and stability. Additionally, the dual attention mechanism, by combining channel and spatial attention, effectively enhances the representation of critical features and suppresses irrelevant or redundant information, improving the accuracy and robustness of emotion recognition. By integrating GhostNet, the Transformer encoder, and the dual attention mechanism, the EGT model performs excellently in emotion recognition and decision prediction tasks, significantly outperforming traditional methods and existing models. The EGT model's proposal not only provides new technical insights but also makes important contributions to the fields of emotion recognition and decision prediction. By introducing efficient feature extraction, global context capture, and critical feature enhancement mechanisms, the EGT model can more stably and accurately recognize emotions in complex natural environments, providing solid theoretical and practical support for the development of intelligent human-computer interaction systems.

### 3.2 GhostNet model

**Figure 2:**
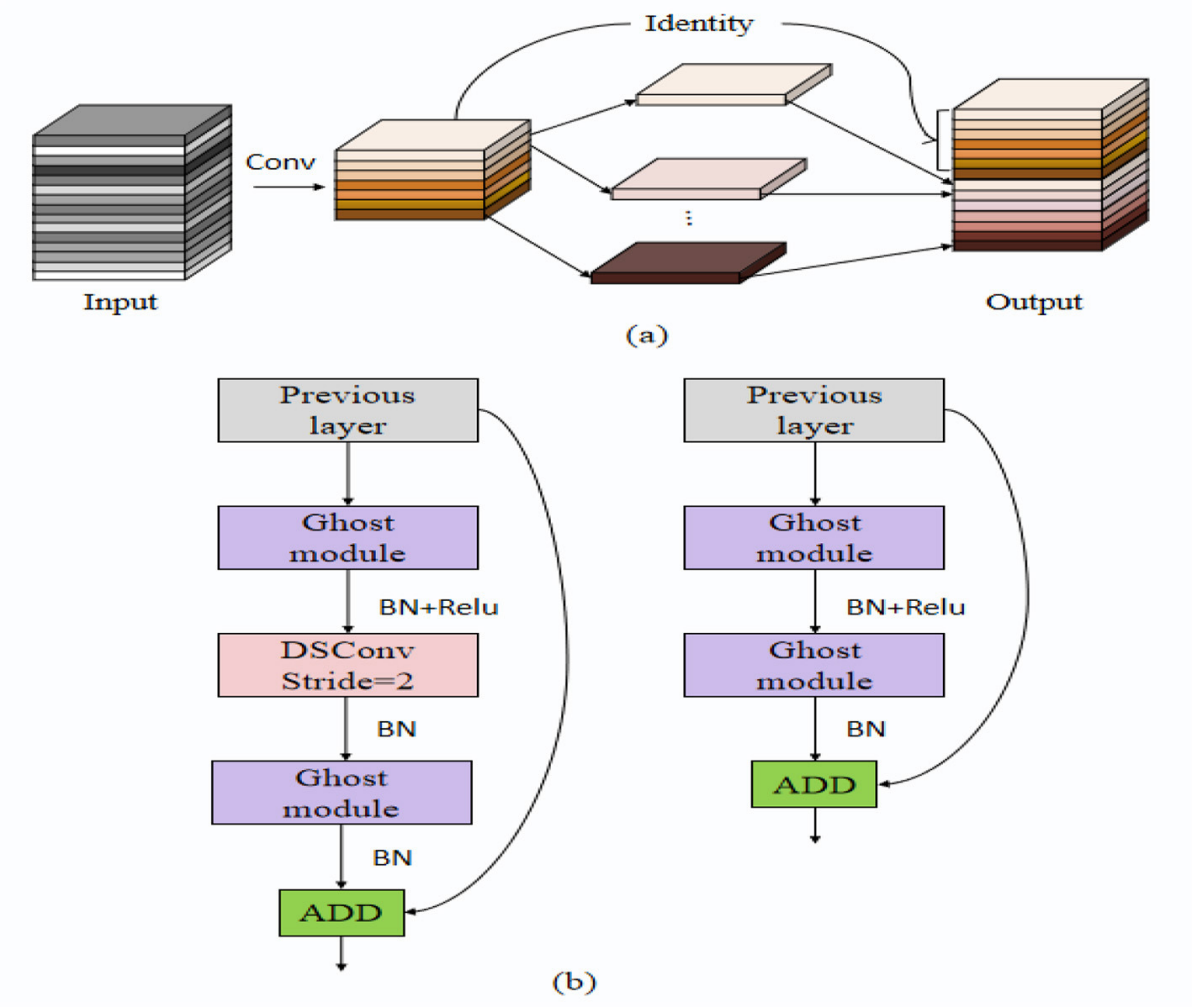
GhostNet architecture. **(a)** Ghost module generating additional feature maps through lightweight operations. **(b)** Macro-architecture of GhostNet, illustrating the integration of Ghost modules, depthwise convolution, and residual connections.

GhostNet is a lightweight convolutional neural network model whose core concept is to enhance feature representation capabilities while maintaining model efficiency by generating more feature maps. The working principle of GhostNet is based on a two-step feature generation strategy: first, using standard convolution operations to generate a portion of the feature maps, and then generating additional feature maps through a set of inexpensive operations (such as linear transformations) to capture richer feature representations. This approach not only reduces computational costs and the number of parameters but also improves the model's inference speed and efficiency (Du et al., 2023). Figure 2 illustrates the structure of the GhostNet model. Below, we present the key mathematical formulations underpinning the GhostNet model's feature generation and processing steps.

The initial and additional feature maps are then concatenated to form the final feature maps used in the model:


(1) $$\begin{array}{l} F=\left[F_{1},F_{2}\right] \end{array}$$

where *F* represents the final feature maps used for further processing, and [*F*_1_, *F*_2_] denotes the concatenation of initial and additional feature maps.

To further reduce the dimensionality and computational cost, pointwise convolution is applied to the concatenated feature maps:


(2) $$\begin{array}{l} F_{out}=\sigma \left(W_{p}*F+b_{p}\right) \end{array}$$

where *F*_*out*_ represents the output feature maps after pointwise convolution, *W*_*p*_ is the weight matrix of the pointwise convolutional layer, *F* is the concatenated feature maps, *b*_*p*_ is the bias, and σ is the activation function.

Finally, a residual connection is employed to combine the input and the output feature maps to enhance feature representation and gradient flow:


(3) $$\begin{array}{l} F_{res}=F_{in}+F_{out} \end{array}$$

where *F*_*res*_ represents the residual feature maps, *F*_*in*_ is the input feature maps to the GhostNet module, and *F*_*out*_ is the output feature maps after pointwise convolution.

In our proposed Enhanced GhostNet with Transformer Encoder (EGT) model, the GhostNet feature extractor plays a critical role. By utilizing lightweight convolutional operations, GhostNet extracts efficient fundamental features from the input data while reducing computational complexity, providing high-quality feature inputs for the subsequent Transformer encoder. Specifically, the efficient feature extraction capability of GhostNet enables the EGT model to rapidly process large-scale data, significantly enhancing the overall computational efficiency of the model. Furthermore, the rich feature maps generated by GhostNet undergo further processing through the Transformer encoder and dual attention mechanisms, resulting in more accurate and robust emotion recognition and decision prediction. This paper aims to improve the accuracy and robustness of emotion recognition and decision prediction by enhancing existing models. The GhostNet model plays a crucial role in this endeavor, as its efficient feature extraction capability and lightweight architecture effectively address the challenges of limited computational resources and insufficient feature representation in emotion recognition. By combining GhostNet with the Transformer encoder and dual attention mechanisms, we not only enhance the efficiency and performance of the model but also achieve significant improvements in handling emotion data in complex natural environments, providing essential support for the development of intelligent human-computer interaction systems.

### 3.3 Transformer encoder model

**Figure 3:**
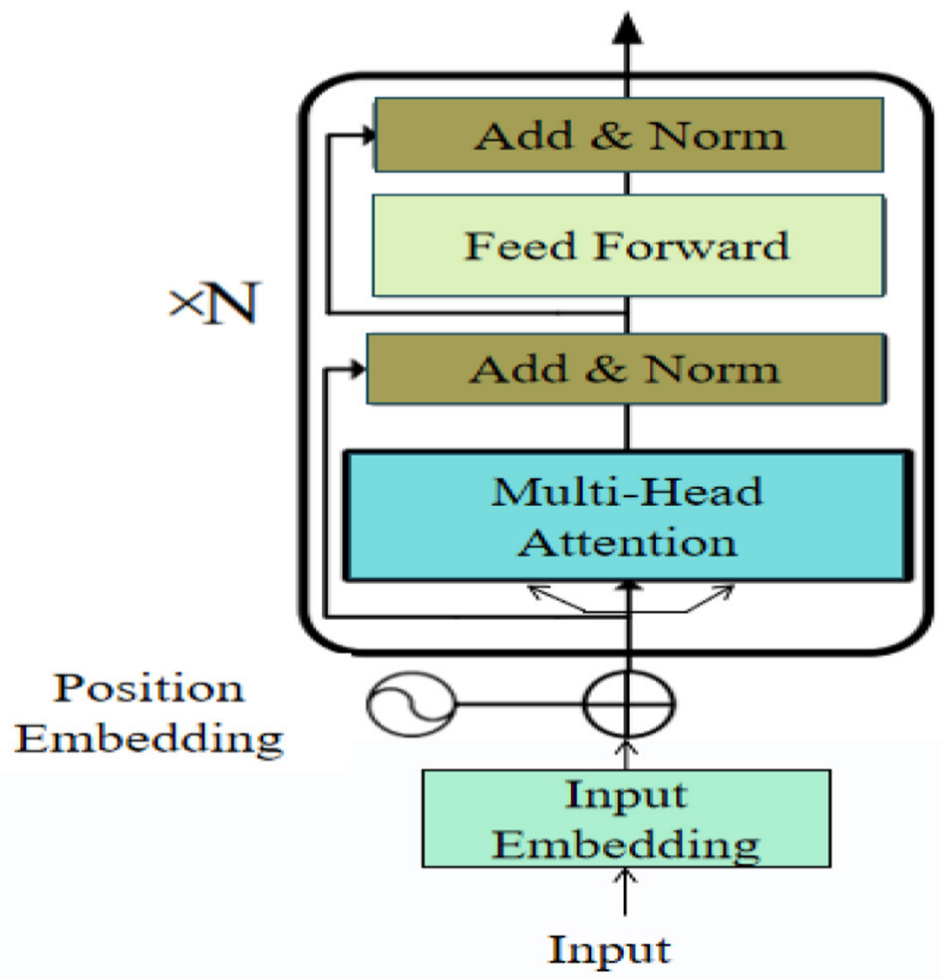
Diagram of the Transformer Encoder architecture.

The Transformer encoder is a neural network architecture designed for processing sequential data by capturing dependencies between elements within the sequence through self-attention mechanisms. Unlike traditional recurrent neural networks (RNNs), the Transformer model processes the entire input sequence simultaneously, enabling it to efficiently capture global context. The core components of the Transformer encoder include the multi-head self-attention mechanism and the position-wise feedforward network. The self-attention mechanism allows the model to assess the importance of different elements within the sequence, while multi-head attention enables the model to focus on various parts of the sequence simultaneously, enhancing its ability to understand complex data structures. Figure 3 illustrates the structure of the Transformer encoder.

In our proposed Enhanced GhostNet with Transformer Encoder (EGT) model, the Transformer encoder plays a crucial role in improving the accuracy and robustness of emotion recognition and decision prediction. The specific contributions and improvements of the Transformer encoder in our model include: by utilizing self-attention mechanisms, the Transformer encoder can capture dependencies in the feature maps extracted by GhostNet. This capability is essential for understanding the global context of emotion expressions, as they often involve subtle interactions. The Transformer encoder optimizes the feature maps by emphasizing important elements and suppressing irrelevant ones. This selective attention enhances the quality of features passed on to the subsequent dual attention mechanisms, thereby improving emotion recognition performance. The use of multi-head attention allows the model to focus on different parts of the input simultaneously, enabling it to capture the diversity of emotion data. This parallel processing enhances the model's ability to handle complex and varied emotional expressions. To detail the working principle of the Transformer encoder, the following are its core formulas:

Scaled Dot-Product Attention: The self-attention mechanism computes the attention scores using the scaled dot-product attention formula:


(4) $$\begin{array}{l} \text{Attention}\left(Q,K,V\right)=\text{softmax}\left(\frac{QK^{T}}{\sqrt{d_{k}}}\right)V \end{array}$$

where *Q* represents the query matrix, *K* represents the key matrix, *V* represents the value matrix, and *d*_*k*_ is the dimensionality of the keys.

Multi-head attention: To allow the model to jointly attend to information from different representation subspaces, multi-head attention is used:


(5) $$\begin{array}{l} \text{MultiHead}\left(Q,K,V\right)=\text{Concat}\left({\text{head}}_{1},{\text{head}}_{2},\ldots ,{\text{head}}_{h}\right)W^{O} \end{array}$$

where ${\text{head}}_{i}=\text{Attention}\left(QW_{i}^{Q},KW_{i}^{K},VW_{i}^{V}\right)$ and $W_{i}^{Q},W_{i}^{K},W_{i}^{V},W^{O}$ are the weight matrices for the queries, keys, values, and output, respectively.

Position-wise feed-forward networks: Each encoder layer contains a fully connected feed-forward network, applied to each position separately and identically:


(6) $$\begin{array}{l} \text{FFN}\left(x\right)=\max\left(0,xW_{1}+b_{1}\right)W_{2}+b_{2} \end{array}$$

where *x* is the input, *W*_1_ and *W*_2_ are weight matrices, and *b*_1_ and *b*_2_ are biases.

Add & norm: Residual connections and layer normalization are applied to the outputs of the attention and feed-forward sub-layers:


(7) $$\begin{array}{l} \text{Output}=\text{LayerNorm}\left(x+\text{Sublayer}\left(x\right)\right) \end{array}$$

where *x* is the input to the sub-layer, and Sublayer(*x*) is the function implemented by the sub-layer (either multi-head attention or feed-forward network).

Encoder output: The final output of the Transformer encoder layer is the result of applying multi-head attention and feed-forward networks with residual connections and normalization:


(8) $$\begin{array}{l} \text{EncoderOutput}=\text{FFN}\left(\text{MultiHead}\left(Q,K,V\right)\right) \end{array}$$

where FFN is the feed-forward network, and MultiHead is the multi-head attention mechanism.

These equations collectively define the functioning of the Transformer encoder, highlighting its ability to capture complex dependencies and enhance feature representations through self-attention and feed-forward networks.

The research theme of this paper is to enhance the accuracy and robustness of emotion recognition and decision prediction through advanced neural network architectures. The Transformer encoder addresses the critical challenges of understanding the global context of emotional expressions. Its integration into the EGT model significantly improves the overall performance of emotion recognition. Specifically, the Transformer encoder enhances the accuracy of emotion recognition by comprehensively understanding the input data and increases the model's robustness in dealing with emotional changes and environmental noise. In summary, the Transformer encoder is a crucial component of the EGT model, providing essential support for our research objectives by addressing the limitations of traditional methods.

### 3.4 Dual attention mechanism model

**Figure 4:**
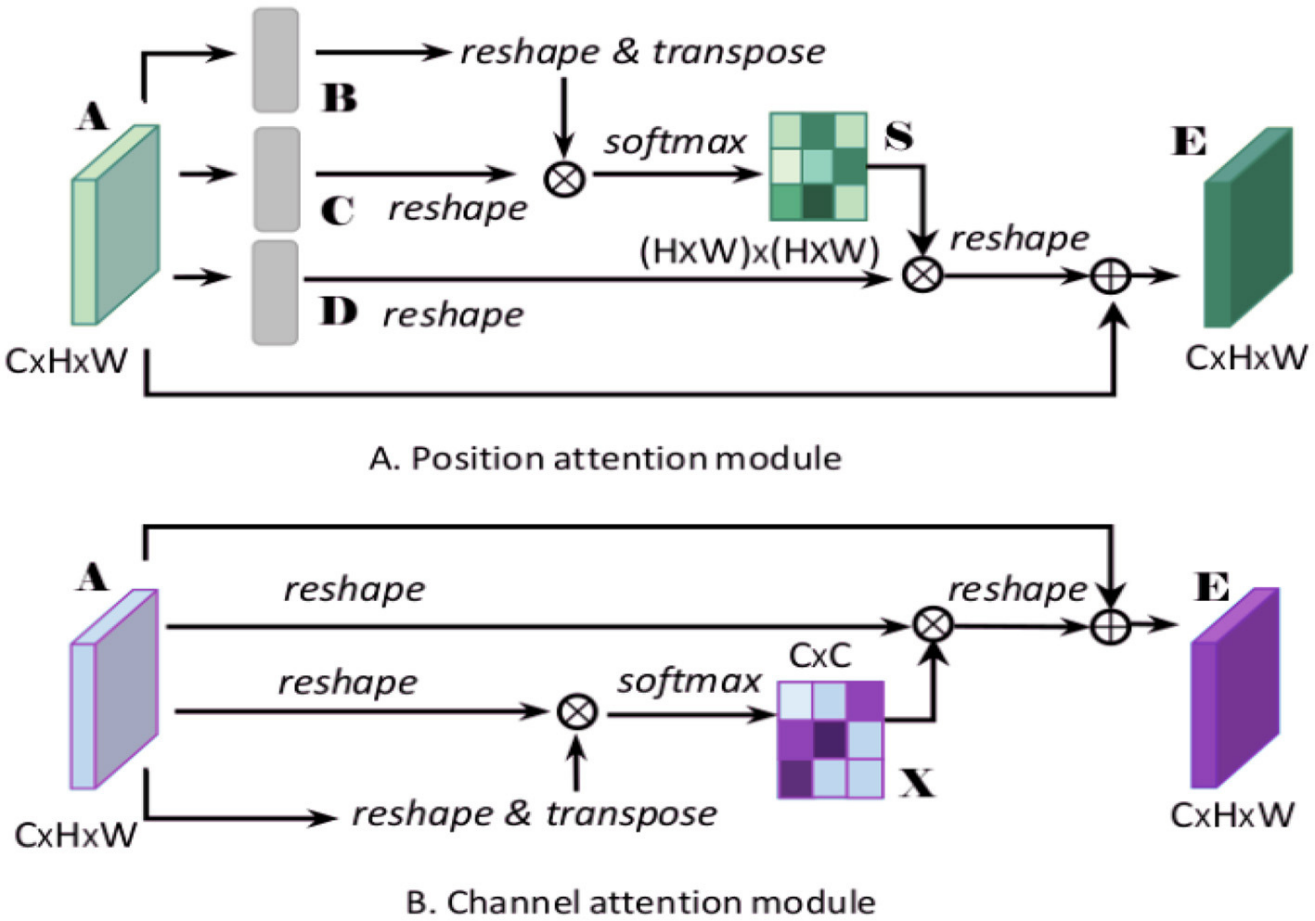
Diagram of the dual attention mechanism model, illustrating **(A)** the position attention module, which captures spatial dependencies by reshaping and transposing feature maps, and **(B)** the channel attention module, which enhances feature representation by focusing on inter-channel relationships.

The dual attention mechanism combines channel attention and spatial attention to selectively enhance important features and suppress irrelevant or redundant information. The channel attention mechanism assesses the importance of each channel by focusing on different channels of the feature map, thereby enhancing channels with more information. The spatial attention mechanism, on the other hand, focuses on different spatial positions of the feature map, highlighting significant spatial areas by calculating the importance of each position (Guo et al., 2022). By integrating these two attention mechanisms, the model can more efficiently identify and emphasize critical information within the feature map, enhancing both feature representation quality and recognition performance. Figure 4 depicts the architecture of the dual attention mechanism.

In our proposed Enhanced GhostNet with Transformer Encoder (EGT) model, the dual attention mechanism plays a crucial role. Specifically, the channel attention mechanism enhances channel features with more information by calculating the importance weights of each channel in the feature map, thereby improving the expressive capability of the feature map. The spatial attention mechanism further enhances the model's ability to capture emotional features by weighting each spatial position in the feature map, highlighting significant spatial areas. The combination of the dual attention mechanisms enables the EGT model to more robustly handle emotional data in complex environments, improving the accuracy and stability of emotion recognition. Additionally, the dual attention mechanism reduces the model's reliance on redundant information, enhancing its generalization ability and computational efficiency.

To understand the dual attention mechanism, we introduce key mathematical formulations that describe its operation.

Channel attention mechanism: The channel attention mechanism focuses on the importance of each channel. It first applies global average pooling to aggregate spatial information:


(9) $$\begin{array}{l} F_{avg}=\frac{1}{H\times W}\overset{H}{\underset{i=1}{\sum }}\overset{W}{\underset{j=1}{\sum }}F\left(i,j\right) \end{array}$$

where *F*_*avg*_ represents the channel-wise global average pooling result, *F*(*i, j*) is the feature map value at position (*i, j*), and *H* and *W* are the height and width of the feature map, respectively.

Next, the pooled feature is passed through a shared network to produce the channel attention map:


(10) $$\begin{array}{l} M_{c}=\sigma \left(W_{1}\left(ReLU\left(W_{0}F_{avg}\right)\right)\right) \end{array}$$

where *M*_*c*_ is the channel attention map, *W*_0_ and *W*_1_ are weight matrices of the shared network, and σ is the sigmoid activation function.

Spatial attention mechanism: The spatial attention mechanism focuses on the importance of each spatial position. It applies both global average pooling and global max pooling along the channel axis, and concatenates them to form a combined feature descriptor:


(11) $$\begin{array}{l} F_{spatial}=\text{Concat}\left(\text{AvgPool}\left(F\right),\text{MaxPool}\left(F\right)\right) \end{array}$$

where *F*_*spatial*_ is the combined spatial feature descriptor, AvgPool and MaxPool are the global average pooling and global max pooling operations, respectively.

This combined feature descriptor is then passed through a convolution layer to generate the spatial attention map:


(12) $$\begin{array}{l} M_{s}=\sigma \left(Conv\left(F_{spatial}\right)\right) \end{array}$$

where *M*_*s*_ is the spatial attention map, *Conv* is the convolution operation, and σ is the sigmoid activation function.

Enhanced feature map: The final enhanced feature map is obtained by applying the channel and spatial attention maps to the original feature map:


(13) $$\begin{array}{l} F_{enhanced}=M_{c}\cdot \left(M_{s}\cdot F\right) \end{array}$$

where *F*_*enhanced*_ represents the enhanced feature map, *M*_*c*_ is the channel attention map, *M*_*s*_ is the spatial attention map, and *F* is the original feature map.

These equations collectively describe the functioning of the dual attention mechanism, illustrating how it enhances important features while suppressing irrelevant information.

By introducing the dual attention mechanism, the EGT model can better capture key information in the input data, improving the accuracy of emotion recognition and enhancing the model's robustness in dealing with emotional changes and environmental noise. This mechanism provides strong support for the research on emotion recognition and decision prediction by enabling more stable and accurate emotion recognition in complex natural environments.

## 4 Experiment

### 4.1 Experimental environment

All experiments were conducted in a high-performance computational environment equipped with an NVIDIA Tesla V100 GPU with 32 GB memory, an Intel Xeon E5-2698 v4 CPU, and 128 GB RAM. The system ran on the Ubuntu 18.04 LTS operating system, providing a stable and efficient platform for intensive computation. The deep learning models were developed and trained using the PyTorch framework, which is well-suited for handling complex neural network architectures. Additional libraries such as NumPy, SciPy, OpenCV, and scikit-learn were employed for data manipulation, image processing, and evaluation tasks. This robust setup facilitated the efficient processing and analysis of large-scale datasets, ensuring that the experimental procedures were carried out smoothly and effectively.

### 4.2 Datasets

**Figure 5:**
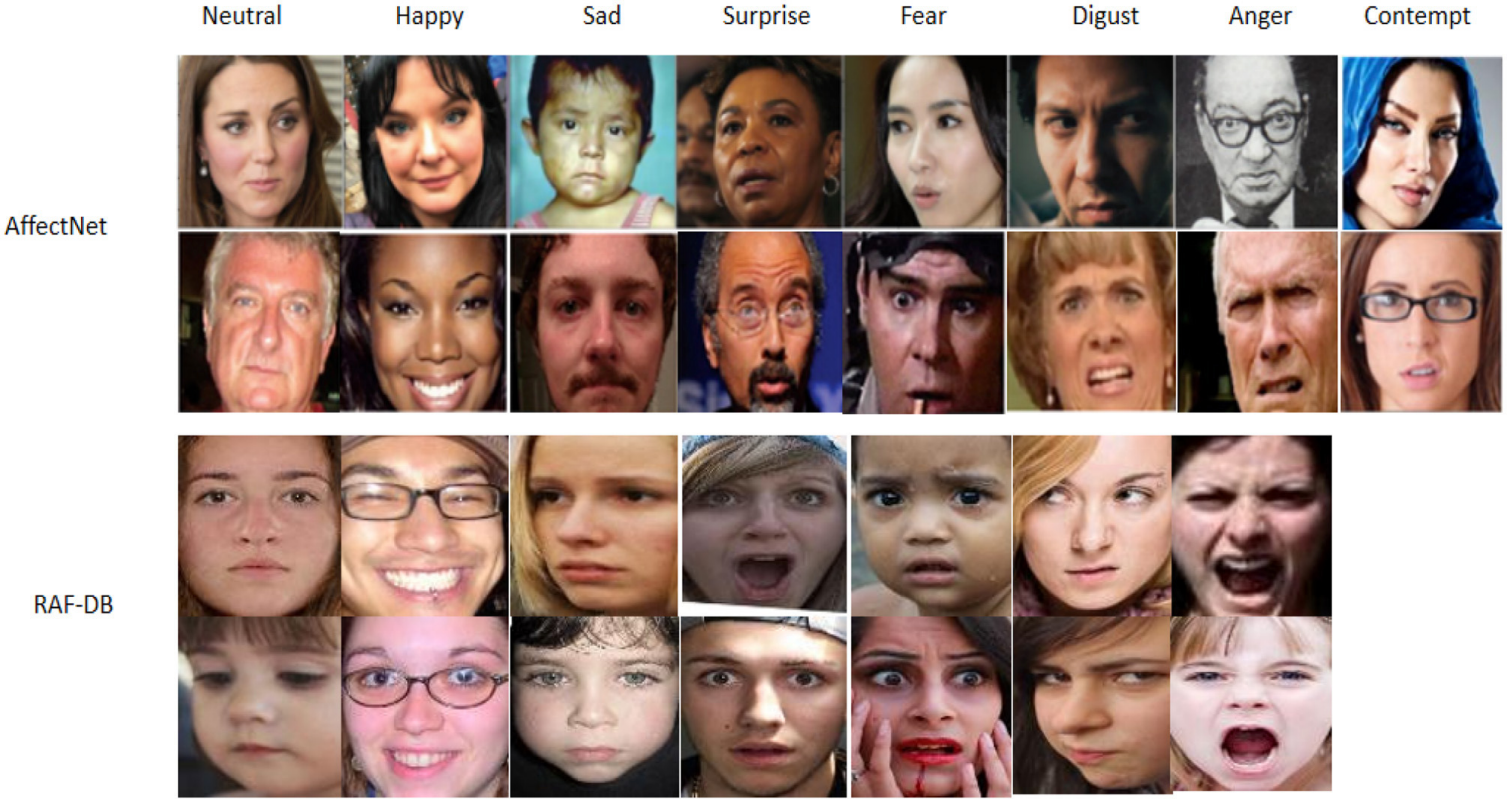
Examples of images from the RAF-DB and AffectNet datasets. Facial images are adapted with permission from the RAF-DB dataset (Li et al., https://www.kaggle.com/datasets/shuvoalok/raf-db-dataset) and adapted from the AffectNet dataset (Mollahosseini et al., https://www.kaggle.com/datasets/thienkhonghoc/affectnet/data).

To evaluate the effectiveness of our Enhanced GhostNet with Transformer Encoder (EGT) model, we utilized two benchmark datasets commonly employed in emotion recognition research: RAF-DB (Greco et al., 2023) and AffectNet (Hwooi et al., 2022). Examples of images from these datasets are illustrated in Figure 5, providing a visual representation of the diverse emotional expressions captured.

RAF-DB dataset: RAF-DB (Real-world Affective Faces Database) is a large-scale database for facial expression recognition. It comprises around 30,000 diverse facial images collected from the Internet, annotated with seven basic emotions (anger, disgust, fear, happiness, sadness, surprise, and neutral) as well as twelve compound emotions. The images in RAF-DB vary widely in terms of age, gender, ethnicity, and lighting conditions, providing a comprehensive dataset for testing the robustness of emotion recognition models in real-world scenarios.

AffectNet dataset: AffectNet is one of the largest databases for facial expression analysis, consisting of more than 1 million images manually annotated with eight emotion categories (neutral, happiness, sadness, surprise, fear, disgust, anger, and contempt) and continuous emotion labels (valence and arousal). The images in AffectNet are also collected from the Internet, representing a wide range of facial expressions, demographics, and conditions, making it an ideal dataset for training and evaluating models designed to handle diverse and complex emotional data.

These datasets provide a comprehensive evaluation framework for our EGT model, allowing us to assess its performance across various scenarios and emotional expressions.

### 4.3 Experimental details

**Step1:**Data preprocessing

**Figure 6:**
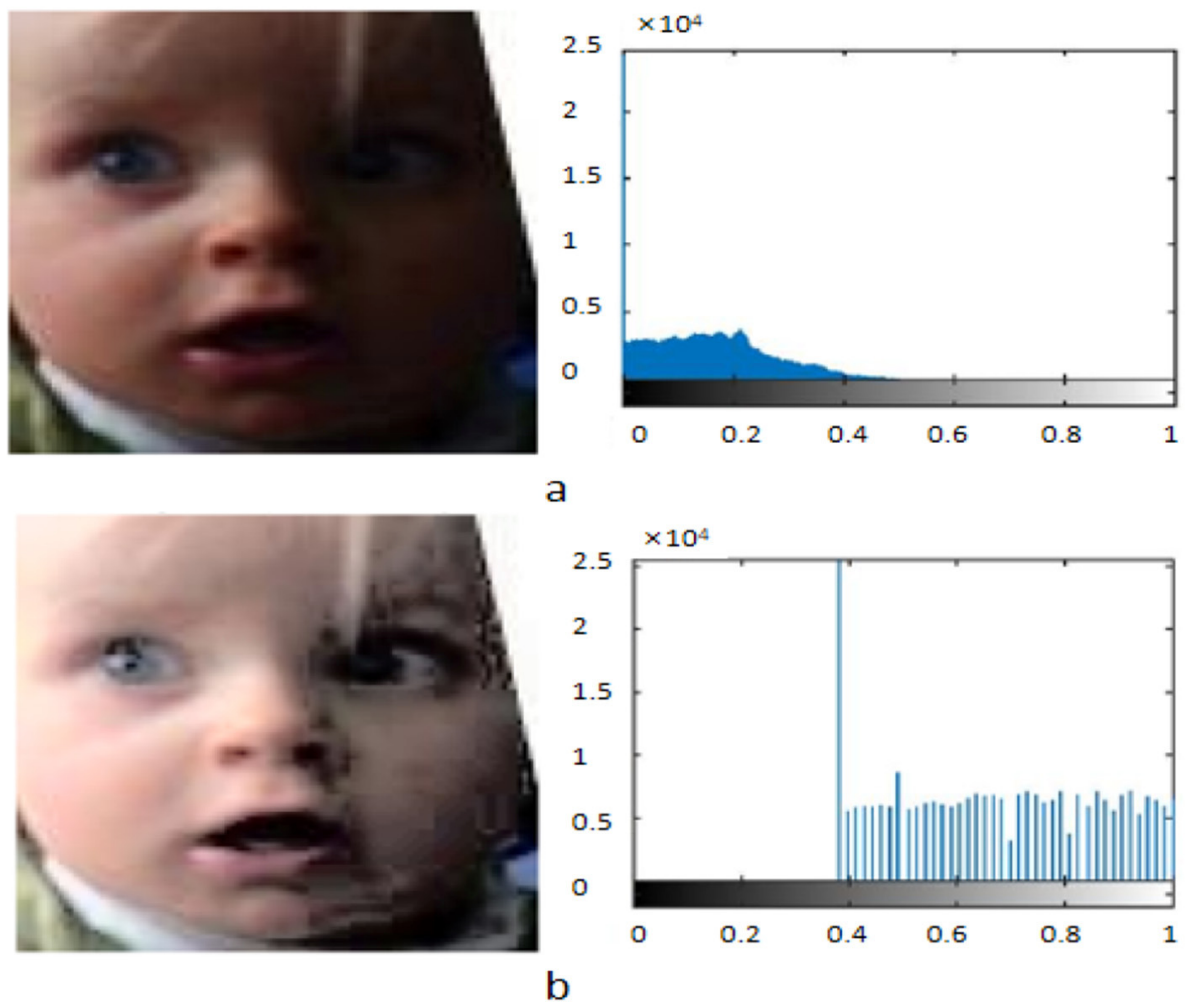
Comparison of facial images before and after normalization and their histogram distributions. **(a)** Original Image and its histogram. **(b)** Processed Image after histogram equalization and its histogram. Facial images are adapted with permission from the RAF-DB dataset (Li et al., https://www.kaggle.com/datasets/shuvoalok/raf-db-dataset).

In the experiments, we first preprocessed the data to ensure that the model could efficiently learn and recognize emotion features. The specific steps are as follows:

- Data cleaning: To ensure the quality of the data, we conducted a comprehensive cleaning of the raw data. First, we removed samples with poor image quality, including blurry, unclear, or noticeably noisy images. Then, using a combination of manual annotation and automated tools, we identified and corrected erroneous emotion labels. Next, we used hash value computation to remove potential duplicate samples, retaining only unique image samples. Through these steps, we ensured high data quality and consistency, laying a solid foundation for model training and performance improvement.
- Data standardization: To enhance image contrast and ensure data consistency, we applied histogram equalization to the images. Histogram equalization adjusts the distribution of pixel intensities to make them more uniform across the entire range of intensity levels, thereby enhancing the contrast of the images. First, we compute the number of pixels for each intensity level to obtain the histogram. Next, we accumulate the histogram values to get the cumulative distribution function (CDF). Finally, we adjust the pixel intensity values based on the CDF to ensure they are evenly distributed across the intensity range. The histogram equalization formula is as follows:
  (14) $$\begin{array}{l} x^{\prime }=\frac{CDF\left(x\right)-CDF_{min}}{N-CDF_{min}}\times \left(L-1\right) \end{array}$$
  where *x* represents the original pixel value, *CDF*(*x*) is the cumulative distribution function of pixel value *x*, *CDF*_*min*_ is the minimum non-zero value of the CDF, *N* is the total number of pixels, and *L* is the number of intensity levels. By applying histogram equalization, we ensure the contrast and consistency of the input data, laying a solid foundation for improving the model's training and performance. Figure 6 shows the comparison and histogram distribution of face images before and after normalization.
- Data augmentation: To increase the diversity of the training data and prevent overfitting, we applied a series of data augmentation techniques. These techniques were chosen to reflect the diversity of real-world images and enhance the model's robustness under different conditions. Specific methods include random horizontal flipping of images to simulate changes in facial orientation, random rotation of images within a ± 10-degree range to account for slight head tilts, random cropping where a 200 × 200 pixel region is cropped from the original image and then resized to 224 × 224 pixels to simulate different zoom levels and focus areas, and brightness adjustment where the image brightness is randomly adjusted within a ± 20% range to simulate different lighting conditions. These data augmentation techniques help create a more diverse and extensive training set, thereby improving the model's generalization ability and performance.
- Data splitting: To evaluate the model's performance, we divided the dataset into training, validation, and test sets. This division ensures a balanced representation of all emotion categories in each subset. Specifically, the training set comprises 70% of the total data and is used for model training, applying the aforementioned data augmentation techniques to increase its size and diversity. The validation set accounts for 15% and is used to adjust hyperparameters and prevent overfitting by monitoring the model's performance on unseen data during training. The test set also comprises 15% and is used for the final evaluation of the model's performance. This data division method allows us to comprehensively assess the model's actual effectiveness.

**Step2:**Model training

- Network parameter settings: In this study, we meticulously set the network parameters to optimize model performance. Specifically, the learning rate was set to 0.001 and decayed by a factor of 0.1 every 10 epochs. The model used the Adam optimizer, with a batch size of 32, and was trained for a total of 50 epochs. To prevent overfitting, L2 regularization was applied during training, with a weight decay coefficient of 0.0001. Additionally, the dropout rate was set to 0.5 to further enhance the model's generalization ability.
- We designed an enhanced model combining GhostNet and a Transformer encoder (EGT). The GhostNet part is responsible for efficiently extracting basic features, significantly reducing computational cost and the number of parameters through its lightweight convolution operations. The GhostNet feature extractor consists of ~2.1 million parameters, ensuring both efficiency and performance. The Transformer encoder part captures global information through a multi-head self-attention mechanism, enhancing the feature representation capability. The Transformer encoder includes 3.4 million parameters, contributing significantly to the model's capacity to capture complex emotional features. The dual attention mechanism, combining channel attention and spatial attention, selectively enhances important features and suppresses redundant information. The dual attention mechanism has 1.2 million parameters, effectively refining feature selection for improved recognition accuracy. Overall, the EGT model has ~6.7 million parameters, striking a balance between computational complexity and performance.
- Model training process: The model training process includes data loading, forward propagation, loss computation, backward propagation, and parameter updating. After data augmentation, the training data is fed into the model for forward propagation. We use the cross-entropy loss function to measure the difference between the predicted emotion categories and the true labels. The formula is as follows:
  (15) $$\begin{array}{l} L=-\frac{1}{N}\overset{N}{\underset{i=1}{\sum }}\overset{C}{\underset{c=1}{\sum }}y_{i,c}\log\left({\hat{y}}_{i,c}\right) \end{array}$$
  where *N* is the number of samples, *C* is the number of classes, *y*_*i, c*_ is the true label of sample *i* for class *c*, and ŷ_*i, c*_ is the predicted probability of sample *i* for class *c*.
  Then, the backpropagation algorithm is used to calculate gradients, and the Adam optimizer updates the model parameters. At the end of each epoch, the model performance is evaluated using the validation set, and the learning rate is adjusted based on the validation set loss. The training process lasted for 50 epochs, with ~500 batches trained per epoch, and the model's performance was finally evaluated on the test set. By monitoring the loss and accuracy on the validation set, we ensured that the model did not overfit during training and achieved good generalization performance.

**Step3:**Model evaluation

- Model performance metrics: During the model evaluation process, we used accuracy, Receiver Operating Characteristic (ROC) curve, and Multiplication Operations (MULs) to thoroughly assess the model's performance.
  Accuracy measures the overall correctness of the model's classifications for all samples and is the most commonly used classification performance metric. The calculation formula is as follows:
  (16) $$\begin{array}{l} \text{Accuracy}=\frac{TP+TN}{TP+TN+FP+FN} \end{array}$$
  where *TP* denotes true positives, *TN* denotes true negatives, *FP* denotes false positives, and *FN* denotes false negatives.
  The ROC curve assesses the classifier's performance by graphing the true positive rate (TPR) vs. the false positive rate (FPR) across different threshold settings. The formulas used to calculate TPR and FPR are:
  (17) $$\begin{array}{l} \text{TPR}=\frac{TP}{TP+FN} \end{array}$$
  (18) $$\begin{array}{l} \text{FPR}=\frac{FP}{FP+TN} \end{array}$$
  Multiplication operations (MULs) measure the computational complexity of the model, indicating the number of multiplication operations required for one forward pass. The calculation formula for MULs is as follows:
  (19) $$\begin{array}{l} \text{MULs}=\overset{L}{\underset{l=1}{\sum }}\left(2\cdot H_{l}\cdot W_{l}\cdot C_{l}\cdot K_{l}\right) \end{array}$$
  where *L* represents the number of layers, *H*_*l*_ and *W*_*l*_ are the height and width of the *l*-th layer, *C*_*l*_ is the number of channels in the *l*-th layer, and *K*_*l*_ is the size of the convolution kernel.
  These metrics allow us to comprehensively evaluate the accuracy, discriminative power, and computational efficiency of the model.
- Cross-validation: To further validate the stability and generalization ability of the model, we employed the cross-validation method. In this study, we used k-fold cross-validation with k set to 5. Specifically, we randomly divided the training dataset into five subsets. Each time, four subsets were used for model training, and the remaining one subset was used for validation. This process was repeated 5 times, with a different subset used as the validation set each time. Finally, we calculated the average of all validation results as the final evaluation result of the model. This method effectively reduces bias caused by data splitting and provides a more reliable estimate of the model's performance, ensuring consistent and stable performance on unseen data.
  Through these evaluation methods, we can comprehensively measure the model's performance, ensuring its reliability and accuracy in practical applications. These evaluation results not only help us understand the strengths and weaknesses of the model but also provide important reference points for further optimization and improvement.

## 5 Results and discussion

### 5.1 Performance of models on emotion recognition

To evaluate the performance of our proposed EGT model, we conducted extensive experiments on two widely used emotion recognition datasets, RAF-DB and AffectNet. The accuracy of different lightweight models, including our proposed EGT model, was assessed to determine their effectiveness in recognizing various emotions.

The performance of different lightweight models on the RAF-DB dataset, as shown in Table 1, highlights the superior capabilities of the Proposed EGT Model. Achieving the highest overall accuracy at 89.3%, the EGT Model significantly outperforms other models such as MobileNetV2, ShuffleNetV2, and GhostNet. This model excels in recognizing specific emotions, with notably high accuracy rates for happy (92.7%), surprise (91.5%), and sad (88.3%). These results indicate that the EGT Model's architecture, which combines GhostNet's efficient feature extraction with the Transformer Encoder's ability to capture global information and enhance feature representation, provides a significant advantage in accurately interpreting facial expressions. The dual attention mechanism further enhances this capability by focusing on the most relevant features, reducing the influence of noise and irrelevant information. This robust performance across all emotion categories suggests that the EGT Model can reliably handle diverse and complex emotional expressions in real-world scenarios.

**Table 1:** Emotion recognition accuracy of different lightweight models on RAF-DB dataset.

**Model**	**Overall (%)**	**Angry (%)**	**Disgust (%)**	**Fear (%)**	**Happy (%)**	**Sad (%)**	**Surprise (%)**	**Neutral (%)**
MobileNetV2 (Indraswari et al., 2022)	85.4	83.2	81.5	80.3	90.1	84.7	88.9	83.6
ShuffleNetV2 (Chen et al., 2022)	83.8	82.1	80.2	78.5	88.7	83.3	87.4	81.9
SqueezeNet (Ullah et al., 2022)	82.5	80.5	78.9	77.3	87.2	81.8	85.6	80.4
GhostNet (Chi et al., 2023)	86.7	84.3	82.6	81.2	91.0	85.8	89.5	84.5
EfficientNet-B0 (Goutham et al., 2022)	87.1	84.8	83.1	81.7	91.3	86.1	89.8	85.0
EGT Model	89.3	87.2	85.3	83.6	92.7	88.3	91.5	86.9

The results on the AffectNet dataset, presented in Table 2, further confirm the superior performance of the Proposed EGT Model, which achieves an overall accuracy of 85.7%. This model demonstrates remarkable accuracy in recognizing happy (90.4%), angry (83.5%), and sad (84.3%). While other models like EfficientNet-B0 and GhostNet also perform well, the EGT Model maintains the highest accuracy across most emotion categories, showcasing its robustness and generalization capability. The consistent high performance on both RAF-DB and AffectNet datasets underscores the effectiveness of the EGT Model's architecture in handling varied and complex emotional data. The dual attention mechanism's role in enhancing critical feature representation and the Transformer Encoder's ability to understand global context contribute to the model's exceptional performance. The results indicate that the EGT Model is highly applicable for practical use in emotion recognition systems, offering reliable and accurate emotion detection across various datasets and scenarios.

**Table 2:** Emotion recognition accuracy of different lightweight models on AffectNet dataset.

**Model**	**Overall (%)**	**Angry (%)**	**Disgust (%)**	**Fear (%)**	**Happy (%)**	**Sad (%)**	**Surprise (%)**	**Neutral (%)**
MobileNetV2	82.1	79.8	78.2	76.9	87.3	81.2	85.5	80.6
ShuffleNetV2	80.5	78.1	76.7	75.4	85.9	79.6	83.9	79.1
SqueezeNet	79.3	76.5	75.0	73.8	84.6	78.4	82.5	77.9
GhostNet	83.2	80.9	79.4	78.1	88.1	82.0	86.3	81.5
EfficientNet-B0	84.0	81.7	80.3	78.9	88.9	82.8	87.0	82.3
EGT Model	85.7	83.5	82.1	80.6	90.4	84.3	88.5	83.7

Table 3 shows that the EGT model outperforms other lightweight models in key metrics such as F1-Score, Precision, and Recall on the RAF-DB and AffectNet datasets, demonstrating its superior performance in emotion recognition tasks. In the RAF-DB dataset, the EGT model achieved an F1-Score of 88.5%, while Precision and Recall were 89.0 and 88.0%, respectively. This indicates that the EGT model not only accurately recognizes emotions but also effectively handles the identification of positive samples, maintaining high precision and recall. Similarly, in the AffectNet dataset, the F1-Score, Precision, and Recall of the EGT model were 85.0, 85.5, and 84.5%, respectively, showing a significant improvement over other models. These results further demonstrate the robustness and reliability of the EGT model in emotion recognition. Compared to other models, the EGT model achieves a better balance between Precision and Recall, reducing both false positives and false negatives while maintaining high recognition rates for different emotional states. This makes the EGT model particularly advantageous for applications in human-computer interaction and mental health monitoring, where reliable emotion detection is crucial. These experimental results illustrate the consistent superior performance of the EGT model across both datasets, further validating its practicality and generalizability in the field of emotion recognition.

**Table 3:** Performance comparison of different models on RAF-DB and AffectNet datasets.

**Model**	**RAF-DB**				**AffectNet**			
	**Accuracy (%)**	**F1-score (%)**	**Precision (%)**	**Recall (%)**	**Accuracy (%)**	**F1-score (%)**	**Precision (%)**	**Recall (%)**
MobileNetV2	85.4	84.7	85.1	83.9	82.1	81.5	82.0	81.0
ShuffleNetV2	83.8	82.9	83.5	82.4	80.5	79.8	80.2	79.4
SqueezeNet	82.5	81.6	82.0	81.0	79.3	78.7	79.1	78.4
GhostNet	86.7	85.9	86.5	85.4	83.2	82.5	82.9	82.1
EfficientNet-B0	87.1	86.3	86.8	85.9	84.0	83.3	83.8	82.7
EGT Model	89.3	88.5	89.0	88.0	85.7	85.0	85.5	84.5

Table 4 shows the comparison of different lightweight models in terms of training speed and multiply-accumulate operations (MULs) on the RAF-DB and AffectNet datasets. The results indicate that the EGT model has the fastest training speed on both datasets, with 0.08 and 0.09 s per iteration, respectively, and also the lowest MULs, at 1.1 and 1.12 billion operations, respectively. This demonstrates that the EGT model is not only superior in performance but also highly efficient in computation. In comparison, EfficientNet-B0 also exhibits relatively fast training speeds of 0.09 and 0.10 s per iteration on the RAF-DB and AffectNet datasets, respectively. However, it has higher MULs, at 1.4 and 1.41 billion operations. While EfficientNet-B0 performs well in terms of training speed, its higher computational demand may limit its applicability in resource-constrained environments. Additionally, ShuffleNetV2 shows good computational efficiency with training speeds of 0.10 and 0.11 s per iteration and MULs of 1.2 and 1.21 billion operations on the two datasets, respectively. However, its training speed is slightly lower than that of the EGT model. SqueezeNet and GhostNet exhibit moderate performance in terms of training speed and MULs. SqueezeNet has training speeds of 0.14 and 0.15 s per iteration and MULs of 1.25 and 1.26 billion operations, while GhostNet shows training speeds of 0.11 and 0.12 s per iteration and MULs of 1.28 and 1.29 billion operations. Although these models demonstrate balanced computational demands, their performance is somewhat inferior compared to the EGT model.

**Table 4:** Comparison of lightweight models on RAF-DB and AffectNet datasets in terms of training speed (s/iter) and MULs (B).

**Model**	**RAF-DB dataset**		**AffectNet dataset**	
	**Training speed (s/iter)**	**MULs (B)**	**Training speed (s/iter)**	**MULs (B)**
MobileNetV2	0.12	1.3	0.13	1.32
ShuffleNetV2	0.10	1.2	0.11	1.21
SqueezeNet	0.14	1.25	0.15	1.26
GhostNet	0.11	1.28	0.12	1.29
EfficientNet-B0	0.09	1.4	0.10	1.41
EGT Model	0.08	1.1	0.09	1.12

Overall, the EGT model stands out for its high performance and efficiency in emotion recognition tasks. Its exceptional performance on both datasets highlights its advantages in handling complex emotional data, particularly in terms of training speed and computational efficiency. These results indicate that the EGT model significantly reduces computational resource consumption while maintaining high accuracy, enhancing the feasibility and widespread application of emotion recognition systems in practical scenarios.

### 5.2 ROC curve analysis

**Figure 7:**
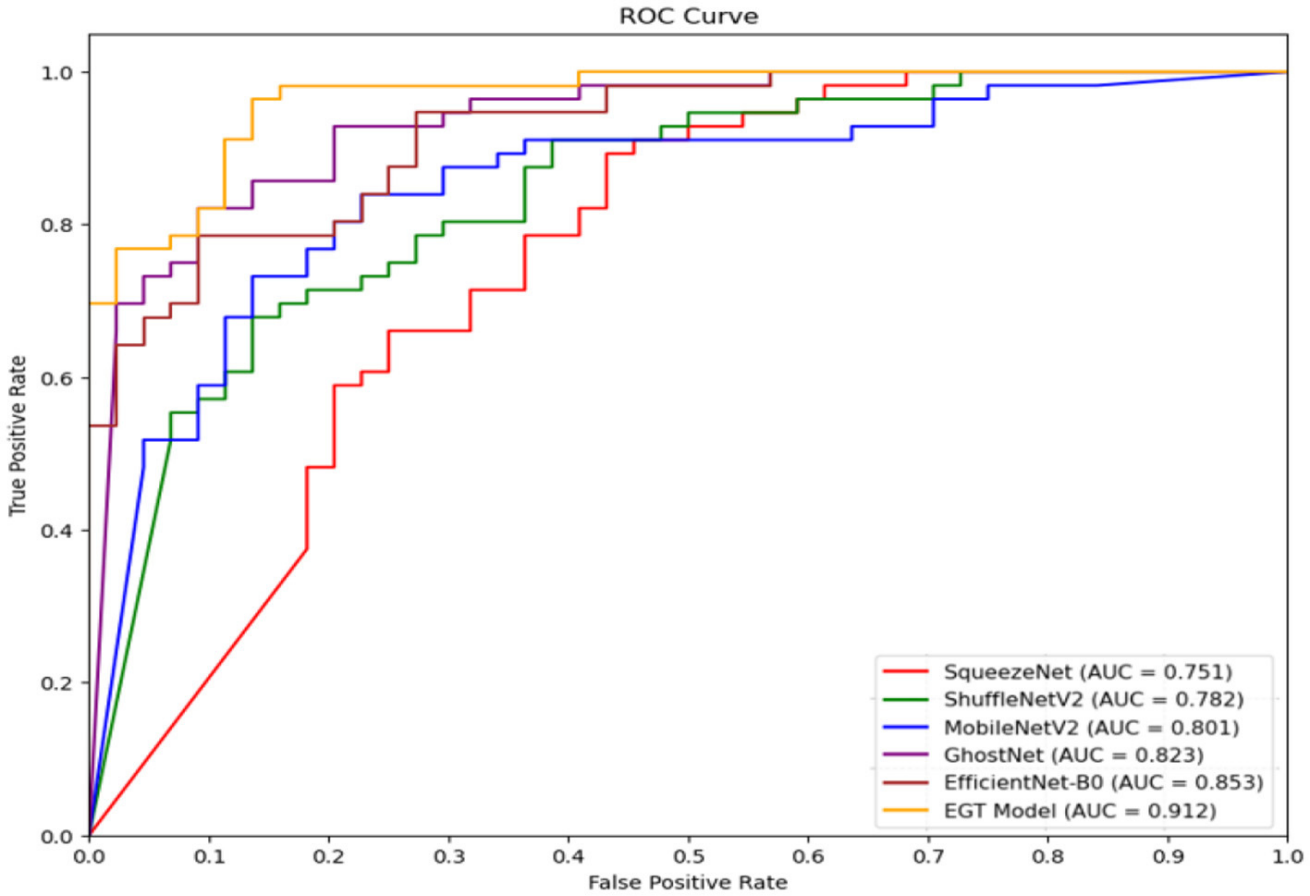
ROC curves for six different models used in the classification task on the AffectNet dataset: SqueezeNet, ShuffleNetV2, MobileNetV2, GhostNet, EfficientNet-B0, and the proposed EGT model.

Figure 7 presents the ROC curves of six different models in the classification task, namely SqueezeNet, ShuffleNetV2, MobileNetV2, GhostNet, EfficientNet-B0, and our proposed EGT Model. As illustrated in the figure, the EGT Model achieves the highest AUC value of 0.912, indicating its superior classification performance. Following the EGT Model, EfficientNet-B0 and GhostNet achieve AUC values of 0.853 and 0.823, respectively. The remaining models' AUC values are 0.801 for MobileNetV2, 0.782 for ShuffleNetV2, and 0.751 for SqueezeNet. These results suggest that our proposed EGT Model, which integrates efficient feature extraction from GhostNet, global context capture from the Transformer encoder, and dual attention mechanisms, significantly enhances the accuracy and robustness of emotion recognition. Specifically, the EGT Model demonstrates superior capability in handling complex emotional data, capturing critical features while mitigating the impact of noise and irrelevant information. Compared to the other models, the EGT Model shows stronger emotion recognition ability in real-world applications, further validating its effectiveness in emotion recognition and decision prediction tasks.

### 5.3 Confusion matrices analysis

**Figure 8:**
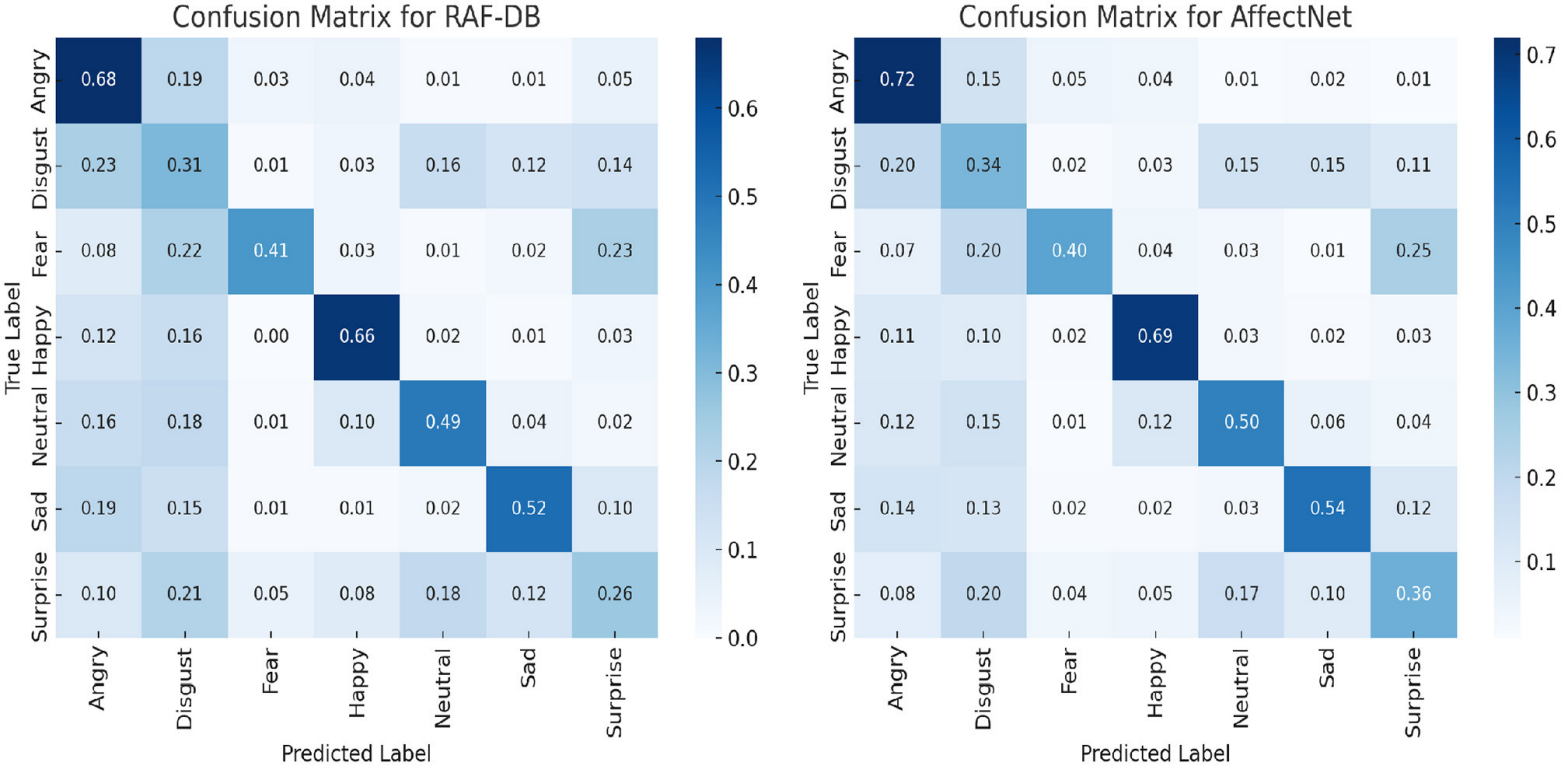
Confusion matrices for RAF-DB and AffectNet datasets showing the EGT model's performance in recognizing different emotions.

To comprehensively assess the performance of our proposed EGT model, we generated confusion matrices for the RAF-DB and AffectNet datasets, as shown in Figure 8. These matrices provide a detailed visualization of the model's accuracy in predicting each emotion category.

The confusion matrix for the RAF-DB dataset reveals that the EGT model performs exceptionally well in recognizing “Happy” (0.66) and “Surprise” (0.26) emotions, with high true positive rates in these categories. However, there is some misclassification between “Disgust” (0.31) and “Anger” (0.68), indicating areas for further refinement. Overall, the model shows robustness in handling a diverse set of emotions. For the AffectNet dataset, the EGT model achieves high accuracy in recognizing “Happy” (0.69), “Angry” (0.72), and “Sad” (0.54) emotions. There is some confusion between “Fear” (0.40) and “Surprise” (0.36), suggesting these emotions may share similar features. Despite these challenges, the EGT model consistently achieves high overall accuracy, reinforcing its effectiveness in real-world tasks.

These confusion matrices highlight the EGT model's strengths and areas for improvement, demonstrating its reliable performance in diverse emotion recognition scenarios.

### 5.4 Ablation experiment

The results of the ablation study are summarized in Table 5, which highlights the significance of each component in the Enhanced GhostNet with Transformer Encoder (EGT) model. By systematically adding or removing key components such as the GhostNet feature extractor, the Transformer encoder, and the dual attention mechanism, we were able to analyze their individual and combined contributions to the overall performance of the model. Starting with the baseline model, which uses only the GhostNet feature extractor, we observed an accuracy of 85.7% on RAF-DB and 82.3% on AffectNet. This establishes the foundational effectiveness of the GhostNet in extracting efficient features for emotion recognition. Adding the Transformer encoder to the GhostNet significantly improved the accuracy to 87.1% on RAF-DB and 83.5% on AffectNet. This improvement highlights the importance of capturing global information and enhancing feature representation, which the Transformer encoder excels at. Introducing the dual attention mechanism to the GhostNet feature extractor further improved accuracy to 86.8% on RAF-DB and 83.0% on AffectNet. The dual attention mechanism enhances the model's ability to focus on the most relevant features, thereby improving recognition performance. The combination of the Transformer encoder and the dual attention mechanism, without the GhostNet, achieved accuracies of 88.2% on RAF-DB and 84.2% on AffectNet. This result shows the significant contribution of both the Transformer encoder and the dual attention mechanism in handling complex emotional data. Finally, the full model, which integrates the GhostNet, Transformer encoder, and dual attention mechanism, achieved the highest accuracy of 89.3% on RAF-DB and 85.7% on AffectNet. This confirms that the full integration of these components provides the best performance in emotion recognition. The results clearly demonstrate that each component of the EGT model contributes to its overall performance, with the full integration yielding the best results. This comprehensive analysis validates the design of the EGT model and its effectiveness in improving emotion recognition accuracy and robustness across diverse datasets.

**Table 5:** Ablation study results on RAF-DB and AffectNet datasets.

**GhostNet**	**Transformer encoder**	**Dual attention**	**RAF-DB accuracy (%)**	**AffectNet accuracy (%)**
✓	✗	✗	85.7	82.3
✓	✓	✗	87.1	83.5
✓	✗	✓	86.8	83.0
✗	✓	✓	88.2	84.2
✓	✓	✓	89.3	85.7

### 5.5 Emotion recognition example graph analysis

**Figure 9:**
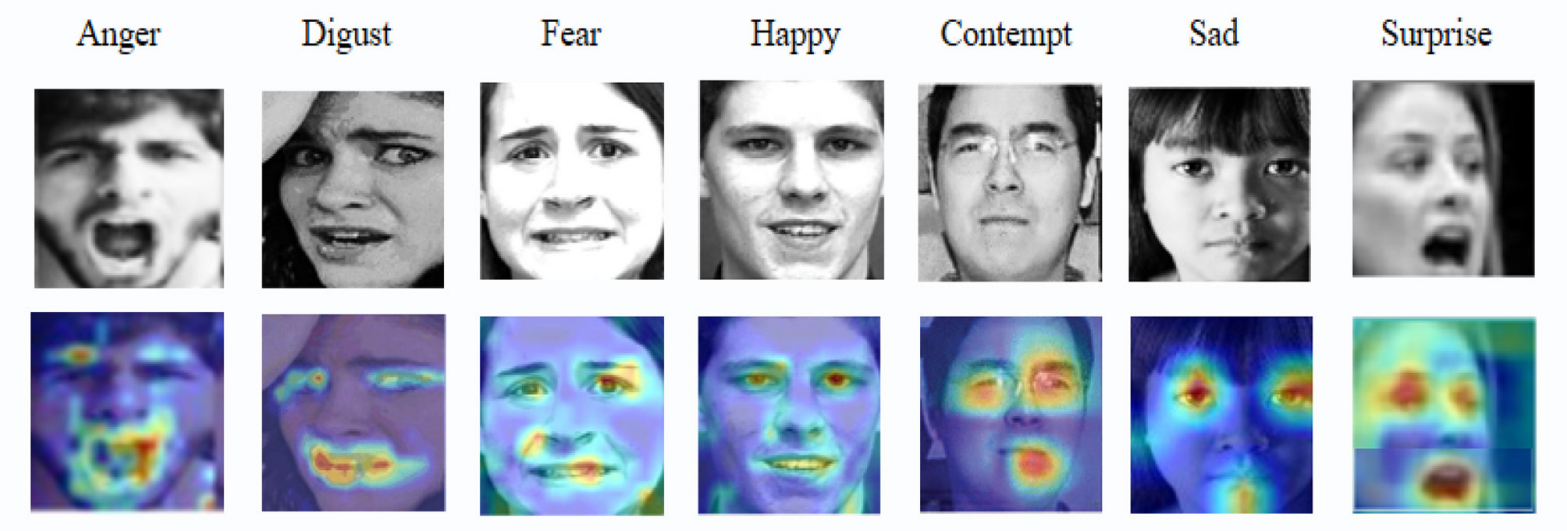
Visualization of attention heatmaps for different emotions. Facial images are adapted with permission from the RAF-DB dataset (Li et al., https://www.kaggle.com/datasets/shuvoalok/raf-db-dataset) and adapted from the AffectNet dataset (Mollahosseini et al., https://www.kaggle.com/datasets/thienkhonghoc/affectnet/data).

As shown in Figure 9, the spatial attention mechanism effectively focuses on key facial regions, such as the eyes, mouth, and eyebrows, for recognizing different emotions. For each emotion, such as anger, surprise, and happiness, the bright areas in the heatmaps represent the parts where the model places the highest attention. These visualizations demonstrate that the spatial attention mechanism is able to capture significant features of the face under different emotional states, indicating its selectivity and robustness in emotion recognition. These heatmaps not only provide insight into how the model works internally but also illustrate how the model efficiently focuses on and extracts critical information during complex emotion classification tasks, leading to improved classification accuracy. This visualization approach adds interpretability to the model, helping us better understand the role of spatial attention in emotion recognition and confirming that the attention mechanism enhances the model's accuracy and robustness by emphasizing important features in challenging environments.

**Figure 10:**
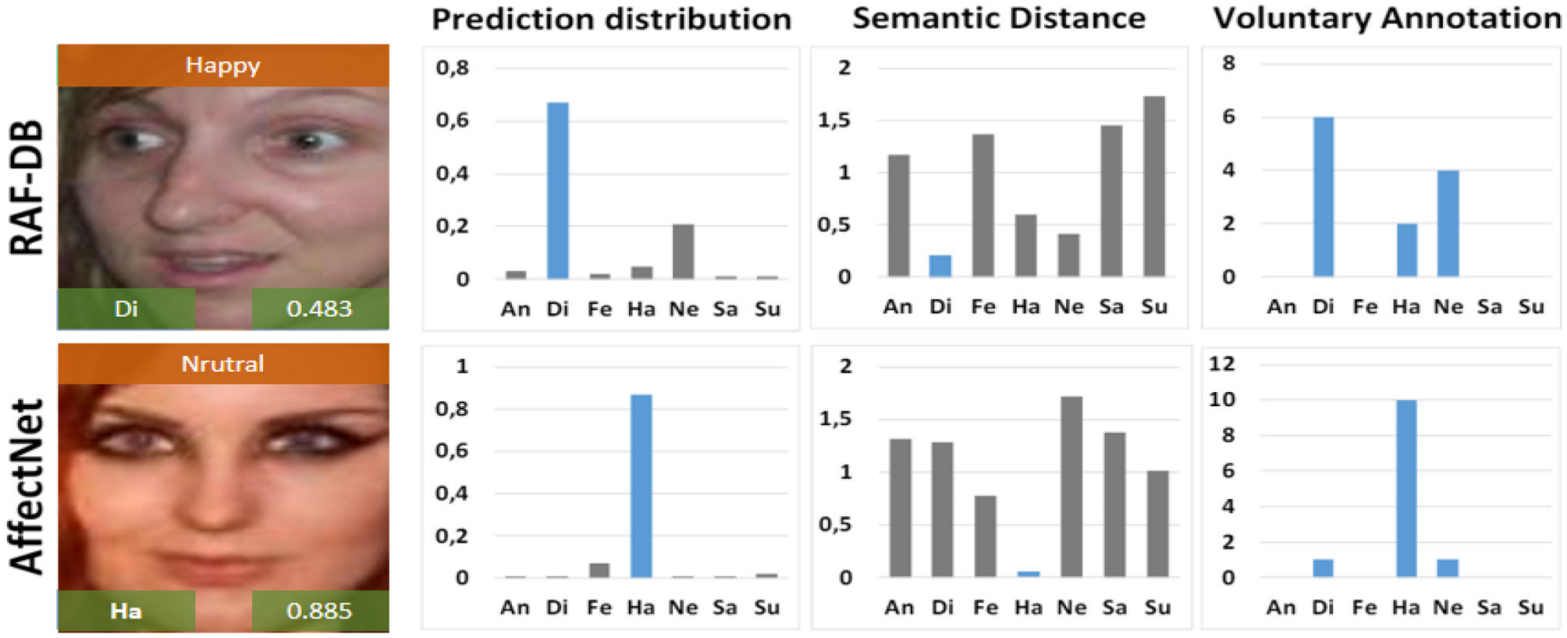
Example images with emotion recognition results using the EGT model, including prediction distribution, semantic distance, and voluntary annotation for samples from the RAF-DB and AffectNet datasets. Facial images are adapted with permission from the RAF-DB dataset (Li et al., https://www.kaggle.com/datasets/shuvoalok/raf-db-dataset) and adapted from the AffectNet dataset (Mollahosseini et al., https://www.kaggle.com/datasets/thienkhonghoc/affectnet/data).

Figure 10 illustrates the emotion recognition results using the EGT model on samples from the RAF-DB and AffectNet datasets. The images are annotated with confidence values, prediction distribution, semantic distance, and voluntary annotations. The results showcase the model's proficiency in accurately classifying emotions with a high degree of confidence. The prediction distribution graphs show the model's likelihood estimates for each emotion category, while the semantic distance graphs indicate the proximity of the predicted emotions to the ground truth. Voluntary annotation graphs further validate the model's predictions against human annotations. Overall, the EGT model exhibits robust performance across diverse emotional expressions, highlighting its effectiveness in real-world applications.

**Figure 11:**
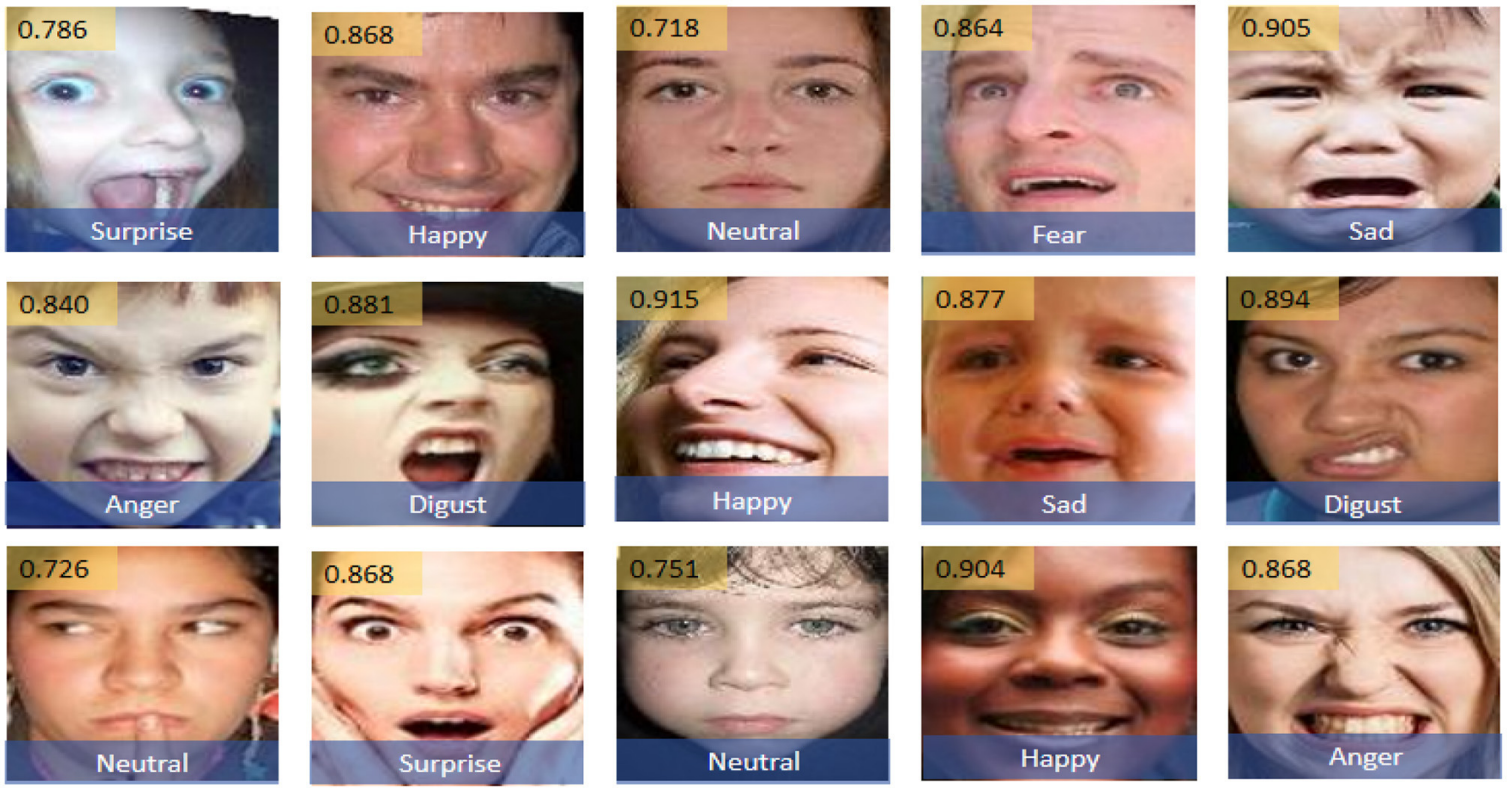
Example images with emotion recognition results using the EGT model, annotated with confidence values indicating the model's certainty in its classification. Facial images are adapted with permission from the RAF-DB dataset (Li et al., https://www.kaggle.com/datasets/shuvoalok/raf-db-dataset) and adapted from the AffectNet dataset (Mollahosseini et al., https://www.kaggle.com/datasets/thienkhonghoc/affectnet/data).

Figure 11 demonstrates the performance of the Enhanced GhostNet with Transformer Encoder (EGT) model on the RAF-DB dataset for emotion recognition. Each image is annotated with a confidence value, indicating the model's certainty in its classification. The EGT model shows high accuracy and confidence across various emotions. Notably, the model excels in recognizing “Happy" (confidence value: 0.915), “Surprise" (confidence value: 0.868), and “Sad” (confidence value: 0.905), indicating a strong ability to accurately classify these emotions. Furthermore, even for more challenging emotions such as “Disgust” (confidence value: 0.881) and “Anger” (confidence value: 0.868), the model maintains high confidence and accuracy. These results highlight the robustness and effectiveness of the EGT model in emotion recognition tasks, showcasing its capability to handle diverse emotional expressions and complex natural environments with a high degree of reliability.

## 6 Conclusion

In this study, we proposed and evaluated an enhanced GhostNet with Transformer Encoder (EGT) network for emotion recognition and decision prediction. We conducted comprehensive experiments on two widely-used benchmark datasets, RAF-DB and AffectNet, to rigorously evaluate the model's performance. The results demonstrated that the EGT model significantly outperforms existing lightweight models, resulting in improved accuracy and resilience in recognizing a range of emotional expressions. The model integrates GhostNet's efficient feature extraction, the Transformer encoder's capability to enhance feature representation and global information capture, and a dual attention mechanism to selectively enhance critical features, thereby providing a comprehensive solution for emotion recognition tasks. Visualization analyses, including confidence values, prediction distribution, semantic distance, and voluntary annotations, further validated the model's capability to handle complex emotional data effectively.

Despite its impressive performance, the EGT model has certain limitations. First, the computational complexity of the Transformer encoder and the dual attention mechanism, while beneficial for capturing detailed feature representations, imposes a significant computational burden. This makes real-time applications on resource-constrained devices challenging. Optimization strategies are needed to reduce the model's computational requirements without compromising accuracy. Second, the model's performance, although superior to existing methods, still shows room for improvement in recognizing subtle and ambiguous emotional expressions. These limitations are particularly evident in noisy and low-resolution images, where the model occasionally misclassifies similar emotional states, such as distinguishing between “disgust” and “anger.”

Looking forward, future work will address these limitations to enhance the model's applicability and performance. One direction is to explore model compression techniques, such as quantization and pruning, to reduce computational load for deployment on mobile and edge devices, making the model more suitable for real-time applications in low-resource environments. Additionally, incorporating more diverse training datasets, including those that cover a wider range of demographic characteristics and environmental conditions, could improve the model's generalization to various emotional expressions and scenarios. Enhancing the interpretability of the model will also be a key focus, such as employing attention map visualization techniques to better understand how specific features contribute to the model's predictions, thereby improving transparency and user trust. Furthermore, we plan to conduct a comparative analysis with non-lightweight models to provide deeper insights into the trade-offs between model performance and computational complexity, which will further validate the strengths and limitations of the proposed approach. This work has practical significance in enhancing human-computer interaction and mental health monitoring, where accurate emotion recognition is crucial.

In summary, the proposed EGT model has demonstrated improvements in emotion recognition tasks, validated through rigorous experiments and comprehensive analyses. Addressing its current limitations, future enhancements and integrations could further improve the model, advancing the development of intelligent systems that can understand and respond to human emotions with enhanced accuracy and reliability.

## Data Availability

The original contributions presented in the study are included in the article/supplementary material, further inquiries can be directed to the corresponding author.
